# Unusual Paraneoplastic Presentation of Cholangiocarcinoma

**DOI:** 10.1155/2015/806835

**Published:** 2015-10-01

**Authors:** Aman Opneja, Sonia Mahajan, Sargam Kapoor, Shanthi Marur, Steve Hoseong Yang, Rebecca Manno

**Affiliations:** ^1^Sinai Hospital of Baltimore, Baltimore, MD 21215, USA; ^2^Washington Hospital Center, Washington, DC 20010, USA; ^3^Johns Hopkins University, Baltimore, MD 21287, USA

## Abstract

*Introduction.* Cutaneous paraneoplastic syndromes are a heterogeneous group of skin manifestations that occur in relation to many known malignancies. Paraneoplastic occurrence of SCLE has been noted but is not commonly reported. SCLE association with cholangiocarcinoma is rare. *Case Presentation.* A 72-year-old man with a history of extrahepatic stage IV cholangiocarcinoma presented with a pruritic rash. Cholangiocarcinoma had been diagnosed three years earlier and was treated. Five months after interruption of his chemotherapy due to a semiurgent surgery, he presented with explosive onset of a new pruritic rash, arthralgias, and lower extremity edema. Physical exam revealed a scaly erythematous rash on his arms, hands, face, neck, legs, and trunk. It was thick and scaly on sun exposed areas. Skin biopsy revealed vacuolar interface dermatitis. Immunofluorescence revealed IgM positive cytoid bodies scattered along the epidermal basement membrane zone. PET-CT scanning revealed metabolically active recurrent disease in peripancreatic and periportal region with hypermetabolic lymph nodes. Oral steroids and new regimen of chemotherapy were started. Rash improved and steroids were tapered off. *Discussion.* Paraneoplastic syndromes demonstrate the complex interaction between the immune system and cancer. Treatment resistant SCLE should raise a suspicion for paraneoplastic etiology.

## 1. Introduction

Paraneoplastic syndromes demonstrate the complex interaction between the immune system and cancer. Presence of a new inflammatory skin condition in a patient with known history of malignancy should raise concern for disease recurrence. Various paraneoplastic cutaneous manifestations of cholangiocarcinoma have been reported, such as alopecia and migratory erythema. However, in the literature, only one case report of subacute cutaneous lupus erythematosus (SCLE) presenting as a paraneoplastic syndrome of cholangiocarcinoma has been reported. We present a case of paraneoplastic SCLE in cholangiocarcinoma and discuss the clues to diagnosis and clinical importance.

## 2. Case Presentation

A 72-year-old man with a history of extrahepatic stage IV cholangiocarcinoma presented with a pruritic rash. Cholangiocarcinoma had been diagnosed three years earlier and was treated with surgical resection followed by radiation and chemotherapy (gemcitabine and capecitabine). Histopathological examination of the surgical specimen including the gall bladder, cystic duct, common bile duct, and hepatic duct revealed moderate to poorly differentiated adenocarcinoma with perineural invasion and free surgical margins. Chemoradiotherapy was completed in the same year but was complicated by recurrent episodes of cholangitis (*E. coli* bacteremia). Two years later, his disease recurred in the liver and chemotherapy was restarted with gemcitabine and cisplatin. Treatment was stopped after three cycles because the patient required a semiurgent abdominal hernia repair.

**Figure 1:**
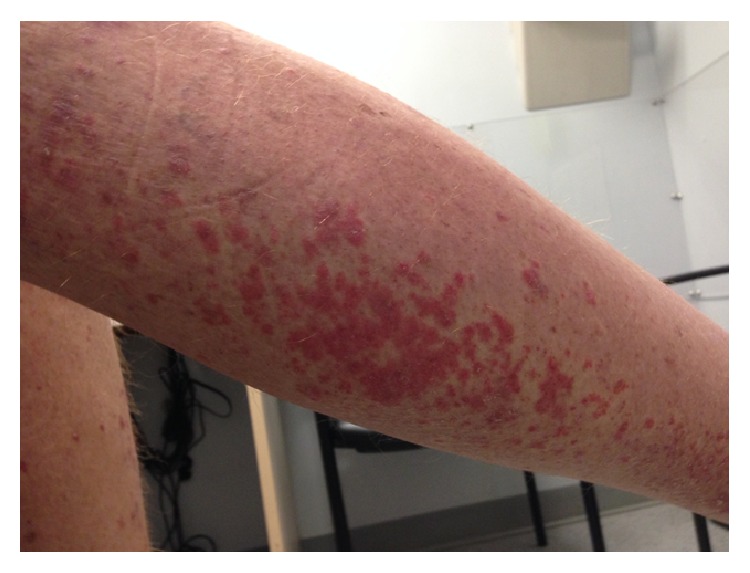
Figure showing pruritic scaly erythematous rash on hands.

**Figure 2:**
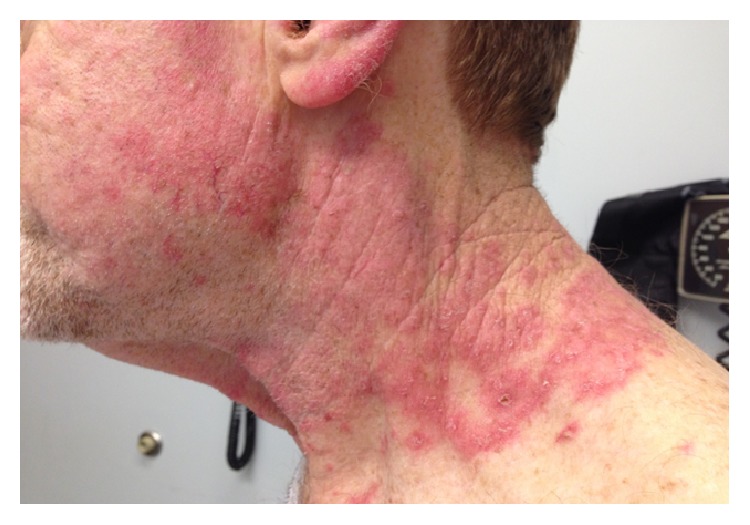
Figure showing spread of erythematous, pruritic rash on neck.

Approximately 5 months after surgery, he experienced the explosive onset of a new pruritic rash, arthralgias, and lower extremity edema. His medications included omeprazole, glucosamine, fish oil, and multivitamin tablet. He had not started any new medications or had any known new cutaneous exposures. Physical exam revealed a scaly erythematous rash on his arms, hands, face, neck, legs, and trunk (Figures 1 and 2). It was thick and scaly on sun exposed areas. His mucous membranes were normal. Digital exam was normal, including nailfold capillary microscopy. Muscle strength was preserved 5/5 and was comparable in all extremities. Respiratory, cardiovascular, and abdominal exams were unremarkable.

**Figure 3:**
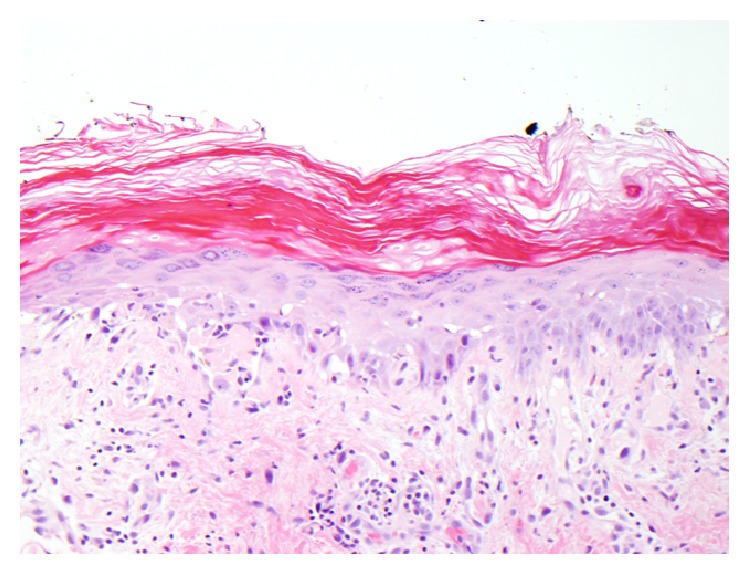
Figure showing skin biopsy of the lesion. Vacuolar alteration of the basal layer and numerous clumped cytoid bodies along the dermoepidermal junction and focally within the spinous layer, stratum corneum, and superficial adnexal epithelium are seen. Within the dermis, there is a superficial and mid perivascular and focally perifollicular inflammatory infiltrate comprised predominantly of lymphocytes with scattered melanophages. Neutrophils and eosinophils are not conspicuous.

Skin biopsy revealed vacuolar interface dermatitis (Figure 3). Immunofluorescence revealed IgM positive cytoid bodies scattered along the epidermal basement membrane zone but no IgA, IgG, fibrin, or C3 deposits.

Blood counts revealed WBC count of 2.47 K/mm^3^, haemoglobin of 11.3 g/dL, and platelet count of 68 K/mm^3^. Liver function test was unremarkable with normal liver enzymes. Electrolytes and kidney function were normal. Immunoglobulin levels were normal. Serology was notable for positive high-titer ANA (homogenous pattern) and negative extractable nuclear antigens. Complement levels were low (C3 96, C4 9 mg/dL) and CA19-9 level was elevated at 2047, which was almost double previous value of 1025 U/mL. Rheumatoid factor levels were 648 units/mL. Antihistone antibodies and dsDNA were negative. Myositis associated and myositis specific antibody panel was negative and CK was normal.

Contrast enhanced CT scan of the abdomen revealed no clear evidence of tumor recurrence. Evidence of prominent porta hepatis and retroperitoneal lymphadenopathy of uncertain significance was noted with mild ascites. Also, note was made of persistent segmental dilatation of the pancreatic duct in the tail region without any evidence of discrete mass. Whole body PET-CT scanning was done which revealed metabolically active recurrent disease adjacent to the postoperative bed in peripancreatic and periportal region with hypermetabolic lymph nodes in pancreaticoduodenal region. However, no abnormal FDG uptake was noted in the skin and subcutaneous tissue or anywhere else in the visualized body.

Oral steroids and new regimen of chemotherapy were started including 5-fluorouracil, oxaliplatin, and irinotecan. His rash showed significant improvement; however, tumour markers remained elevated (CA19-9: 2000).

Slowly, the rash improved and healed. Oral steroids have been tapered off and no exacerbation of rash has been noted to date.

## 3. Discussion

Cutaneous paraneoplastic syndromes are a heterogeneous group of skin manifestations that occur in relation to many known malignancies. They do not have any association with direct tumor invasion or metastases. Diagnosis of paraneoplasia should fulfil the following criteria: (1) both conditions should occur simultaneously (neoplasia and paraneoplasia); (2) both should have a parallel course; (3) skin lesion should not be associated with a genetic syndrome; (4) there is a specific type of neoplasia that occurs with paraneoplasia; (5) dermatosis is rare in the general population; (6) there is a high frequency of association between both conditions [1, 2]. The common skin manifestations of these syndromes include papulosquamous, erythematous, and bullous skin lesions. Cases presenting with erythematous lesions have been linked to dermatomyositis, subacute cutaneous lupus erythematosus (SCLE), erythema gyratum repens, multicentric reticulohistiocytosis, necrolytic migratory erythema, and sweet syndrome.

There are features of our patient's rash which fit well with dermatomyositis, a well-established paraneoplastic condition. The rash of dermatomyositis can be indistinguishable from lupus both clinically and on biopsy, as both have predilection for sun exposed areas (neck, face) and will demonstrate interface dermatitis on biopsy. Immunofluorescence is often negative for immune complex deposition in dermatomyositis, and, similarly, this can be scant or negative in SCLE, as was the case with our patient. However, with other features of an immune complex-mediated process (i.e., hypocomplementemia) and the absence of any muscular findings, Gottron's papules and myositis antibody, we favour a diagnosis of SCLE over dermatomyositis for our patient. However, amyopathic dermatomyositis can have similar presentation. Anti-SSA/Ro antibody was negative in our patient but its association with SCLE is not 100%.

SCLE is characterized by erythematous, nonscarring, papulosquamous, or annular lesions with a photosensitive distribution pattern. Our patient presented with an erythematous, pruritic, plaque-like rash in photodistributed pattern which could be related to allergy, chemotherapeutic drug reaction, infection, immune related pathology, and paraneoplastic or idiopathic etiology. Paraneoplastic etiology was considered in this case, in view of absence of history of contact allergies, systemic clinical signs of infection, or any recent chemotherapeutic drug administration. Presence of photosensitivity, typical serology, supportive histopathology, association with history of cholangiocarcinoma, and raised tumor marker levels led to the diagnosis of paraneoplastic subacute cutaneous lupus erythematosus (p-SCLE). It is also in accordance with the criteria mentioned by McLean; that is, dermatoses arise after the development of malignant tumour and, secondly, both the dermatoses and the tumour follow a parallel course [3].

The first case of p-SCLE was described by Trousseau in 1986 [4]. Since then, approximately 18 cases of p-SCLE have been described and documented in the literature so far [5, 6]. The associated malignancies include predominantly lung and breast carcinomas. One case each has been described in association with hepatic, gastric, uterine, laryngeal, prostate, and oesophageal carcinoma, cholangiocarcinoma, and Hodgkin's lymphoma. Grönhagen et al. reported a twofold increase in malignancy in patients with cutaneous lupus erythematosus. However, no causal relationship was found [7]. To the best of our knowledge, this is only the second case of p-SCLE associated with cholangiocarcinoma. The unique feature about our case is that the photosensitive rash appeared after the completion of treatment and was in relation with uptrending tumor marker levels. The first case described a similar rash which resolved after the tumour debulking surgery [6].

Different mechanisms have been proposed for the development of the paraneoplastic phenomenon; however, the exact mechanism is yet to be defined. Chaudhary et al. give a possible role of stimulatory tumor antigen which shows episodic expression with disease activity, thus reflecting antigen load rather than absolute tumor mass [5]. Ro52 autoantigen is an interferon inducible protein that localizes to the cytoplasm and functions as an E3 ubiquitin ligase, an enzyme that adds ubiquitin molecules to target proteins [8]. Ro52 adds ubiquitin molecule to activated inhibitor of nuclear factor kappa-B kinase subunit beta (IKKB) and helps in downregulating proinflammatory nuclear factor kappa-B (NFKB) signaling [9]. It inhibits inflammation by targeting interferon regulatory factors (IRF) 3 and 7 for ubiquitin mediated degradation [10]. Ro ribonucleoprotein is transferred to the keratinocyte cell membrane with UV exposure and is presented to T lymphocytes by antigen presenting cells in the presence of MHC class II molecules. Antigen specific T-helper cells are activated which trigger the differentiation of B cells to antibody producing plasma cells. With further release of Ro antigen, a self-perpetuating immune response follows and explains the photosensitive nature of rash [11].

Case reports of drug induced SCLE with chemotherapeutic agents like capecitabine and fluorouracil are known [12, 13]. However, the likelihood of such an etiology was low in this case as the patient was on a chemotherapy break from the time of abdominal hernia surgery till the development of rash for a period of 5 months. In summary, the clinical importance of this case is the unusual patient presentation with skin manifestations in association with cancer recurrence, thus highlighting the role of recognizing paraneoplastic syndromes at the time of diagnosis or during the course of malignancy. It was only in the context of this paraneoplastic disease manifestation that a deeper look for disease recurrence was pursued (PET-CT). The radiographic findings of disease recurrence (only appreciated on PET-CT), rising CA19-9, and paraneoplastic phenomenon necessitate treatment that would otherwise have been delayed.
